# Experimental study on the compressive strength of fixed prosthetic restorations on tooth preparations with or without horizontal preparation

**DOI:** 10.25122/jml-2025-0124

**Published:** 2025-07

**Authors:** Lucia Alexandra Drăghici, Raluca Monica Comăneanu, Diana Vrânceanu, Florin Baciu, Mirel Stoian-Albulescu, Anca Monica Dobrescu, Tudor Petru Ionescu, Cherana Gioga

**Affiliations:** 1 Doctoral School of Dental Medicine, Titu Maiorescu University, Bucharest, Romania; 2 Faculty of Dental Medicine, Titu Maiorescu University, Bucharest, Romania; 3 Titu Maiorescu Private Clinical Center of Dental Assistance, Bucharest, Romania; 4 Faculty of Materials Science and Engineering, National University of Science and Technology Politehnica, Bucharest, Romania; 5 Department of Strength Materials, Faculty of Industrial Engineering and Robotics, National University of Science and Technology Politehnica, Bucharest, Romania

**Keywords:** horizontal preparation, vertical preparation, retention, zirconium oxide

## Abstract

Fixed prosthetic restorations, particularly those made entirely of zirconia, are widely used in clinical dental practice for the restoration of natural teeth. Their success largely depends on the design of the tooth preparation, especially the type of finish line, as well as the restoration’s ability to withstand occlusal forces during mastication. Over 12 months, a total of 21 extracted teeth were initially collected, from which six were selected for this study. To achieve the objective of the study, three teeth were prepared with a horizontal finish line (shoulder), and the remaining three were prepared without a defined finish line (vertical preparation). For each prepared tooth, three full-contour zirconia crowns were fabricated. To ensure accurate comparison and standardization of occlusal force application during mechanical testing, all crowns were designed with identical morphology. The force application device used in mechanical testing was digitally designed to replicate the anatomy of the antagonistic teeth corresponding to each tested crown. Samples I and III showed significant structural changes, including horizontal fractures at the cervical level. Samples II, 1, and 2 exhibited no visible damage. The maximum recorded compressive forces at which structural failure occurred varied widely, ranging between 180 N and 2537 N. Consequently, all samples that recorded fracture values below 1000 N were analyzed separately for interpretation. The findings of this experimental study indicate that tooth preparation design significantly influences the compressive strength of monolithic zirconia crowns. Both preparation types—shouldered and shoulderless—demonstrated high resistance values, with a slight advantage observed in favor of the preparations with a defined finish line (shoulder).

## INTRODUCTION

Fixed prosthetic restorations, particularly full-contour zirconia crowns, are frequently used in restorative dental practice. Their clinical success largely depends on the design of tooth preparation—especially the finish line configuration—and on the restoration's ability to withstand occlusal forces during mastication. In vitro, the type of finish line has been shown to influence fracture resistance. A study published in Cureus reported that both heavy chamfer and biologically oriented preparation technique (BOPT) designs generate lower stress levels at the margin for zirconia crowns. Other studies have supported the notion that shoulder-type preparations offer the highest fracture resistance for zirconia restorations [1,2].

A comparative in vitro study that evaluated prominent shoulder preparations, chamfer margins, and biologically oriented designs (heavy chamfer, shoulder, and BOPT) demonstrated that zirconia crowns prepared with a deeper chamfer margin exhibited significantly higher fracture resistance compared to shoulder preparations (*P* = 0.004). No statistically significant difference was found between the chamfer and BOPT designs [3,4].

Furthermore, specialized studies have shown that the fracture resistance of monolithic zirconia crowns significantly decreases as the marginal thickness is reduced; with a margin thickness >1 mm, fracture resistance can exceed ≈1,900 N, while a 0.2 mm reduction may decrease resistance by approximately 300–400 N [5]. In another study, monolithic Y-TZP zirconia crowns placed on vertical (shoulderless) preparations demonstrated extremely high fracture resistance (up to 5,712 N), surpassing those prepared with a 0.8 mm chamfer (≈5,090 N) or a 0.4 mm chamfer (≈4,703 N) [6]. The current literature consistently shows that zirconia and metal-ceramic crowns, when tested on preparations with heavy chamfer, shoulder, or vertical finish lines, demonstrate fracture values ranging between 1,500 and 2,500 N—well above the maximum simulated natural occlusal forces (600–900 N). The choice of finish line type (chamfer vs. shoulder vs. BOPT) significantly influences marginal adaptation, mechanical stability, and the biological response of the gingival tissues.

The present study experimentally investigated the maximum compressive forces that full-contour zirconia crowns can withstand when cemented on abutments prepared with or without a defined finish line. The aim was to determine whether the design of the finish line significantly impacts the durability of the restoration in standardized in vitro conditions, to address gaps in the current literature, and to provide results that are comparable with high-value experimental studies [7].

The objective of this study was to identify the maximum fracture resistance of single-unit zirconia crowns and to evaluate how this is influenced by two different prosthetic preparation techniques, with and without a finish line.

## MATERIAL AND METHODS

**Figure 1:**
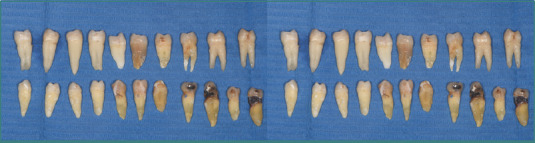
Macroscopic image of the extracted teeth

Over 12 months, a total of 21 extracted premolars (Figure 1), removed for orthodontic and periodontal reasons, were collected. From these, 12 teeth unaffected by carious lesions and presenting similar anatomical features were selected. Subsequently, six of these teeth were prosthetically prepared: three with a finish line (shoulder) and three without a finish line (vertical preparation).

**Figure 2:**
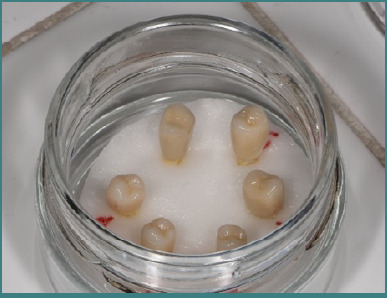
Image showing the method used to maintain the teeth in a hydrated state

Throughout the collection period, the teeth were stored in a saline solution (Figure 2) to prevent dehydration and inhibit biofilm formation on their surfaces.

**Figure 3:**
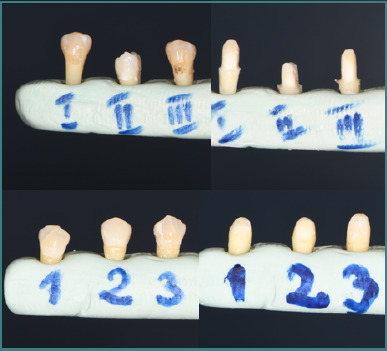
Images of the tooth preparations

To achieve the study objective, three of the six selected teeth were prepared with a finish line, and three without a finish line (Figure 3). For each of these teeth, single-unit full-contour zirconia crowns were fabricated.

**Figure 4:**
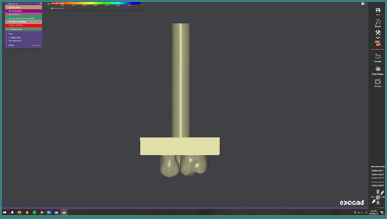
The load-application device in its virtual (digital) design form

To ensure optimal comparability of results and to accurately replicate the contact and force application of antagonistic teeth during mechanical testing, all zirconia crowns were designed with identical morphology. This was made possible through collaboration with the dental laboratory, where the restorations were fabricated. The force-application device used during mechanical testing was digitally designed to match the anatomy of the antagonistic tooth selected for the tests (Figure 4). The zirconia crowns and the metallic abutment simulating the antagonistic teeth subjected to loading were manufactured in collaboration with the 3Dentasoft Dental Laboratory, Bucharest.

**Figure 5:**
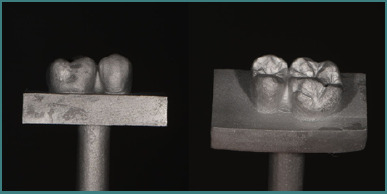
Macroscopic view of the device used for load application during mechanical testing

This device was subsequently manufactured from a high-strength material, selected specifically to withstand the planned mechanical tests without influencing the results in any way. The material chosen was a cobalt-chromium (Co-Cr) alloy, and an image of the fabricated device is presented in Figure 5.

**Figure 6:**
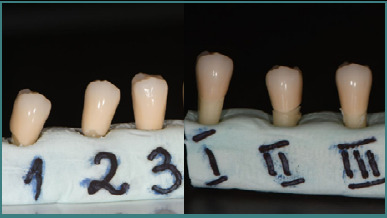
Macroscopic view of dental crowns cemented onto teeth

After fabrication, the dental crowns were permanently cemented with the same glass ionomer luting agent (GC Fuji I; Figure 6). The restored teeth were then stored in saline to prevent dehydration until the next preparation step.

**Figure 7:**
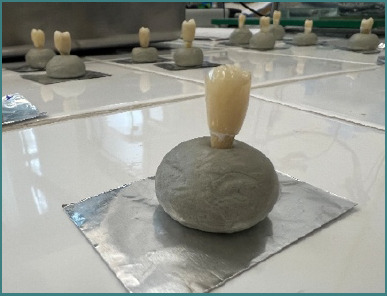
Image showing the fixation of the specimens in epoxy resin

**Figure 8:**
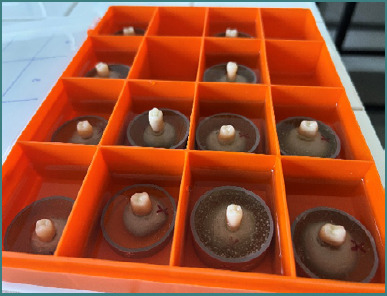
Macroscopic view of a tooth embedded in the support structure used for mechanical testing

In order to perform the mechanical tests, it was necessary to ensure that all prepared teeth with cemented crowns were positioned vertically and supported at their base by a rigid, non-deformable structure with a sufficiently large contact surface. To achieve this, the teeth were initially embedded in an epoxy resin (Figure 7) and subsequently placed into cylindrical molds with a diameter of 40 mm and a height specific to each tooth. These molds were then filled with an acrylic resin (Duracryl). Between each of these steps, the embedded or fixed teeth were continuously stored in saline solution to prevent dehydration (Figure 8).

Compression mechanical tests were performed using an INSTRON 8872 universal testing machine. The tests were recorded with a high-speed camera. For testing, the support containing the embedded teeth was secured to the fixed platform of the testing machine, while the specially designed device for these tests was attached to the movable crosshead. The device applied load to the teeth at a speed of 1.5 mm/min, and the maximum force at which an event occurred—defined as a 10% reduction from the peak recorded force—was recorded.

This study was conducted at the National University of Science and Technology Politehnica Bucharest, Faculty of Materials Science and Engineering.

## RESULTS

The results of the compression tests were recorded in Table 1.

**Table 1:** Maximum forces recorded during the compression tests

Type of preparation	Sample	Compression force (N)
Without a finish line	1	1091
	2	2239
	3	2533
With the finish line	I	2301
	II	405
	III	2537

**Figure 9:**
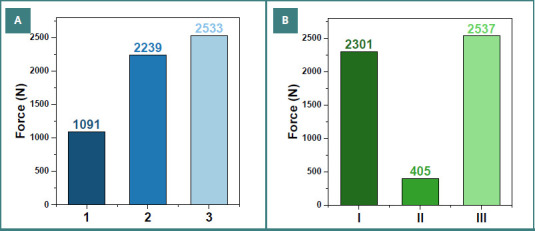
Maximum forces recorded during compression testing of zirconia-crowned teeth. A, Preparations without a finish line (specimens 1–3). B, Preparations with a finish line (specimens I–III).

For more precise visualization, the maximum load values recorded for each specimen type are presented in Figure 9 AB.

**Figure 10:**
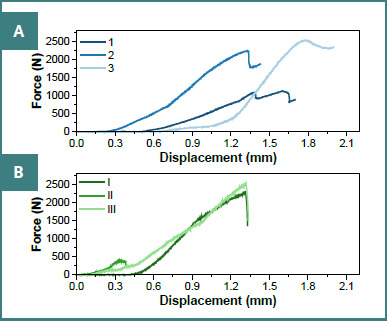
Force–displacement curves for zirconia-crowned teeth. A, Preparations without a finish line (specimens 1–3). B, Preparations with a finish line (specimens I–III).

The forces recorded during compression testing are shown for full-contour zirconia crowns on preparations without a finish line (Figure 10A) and with a finish line (Figure 10B).

**Figure 11:**
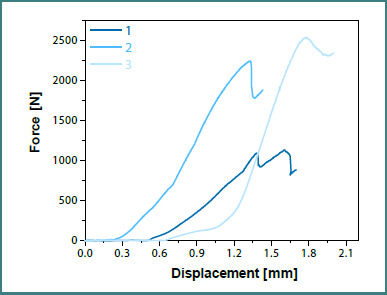
Force-displacement curves for preparations without a finish line

**Figure 12:**
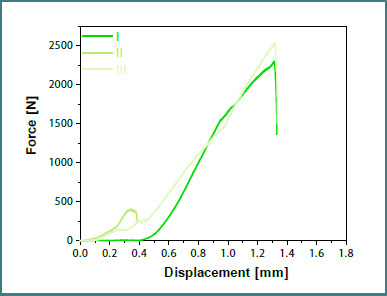
Force-displacement curves for preparations with a finish line

Graphs displaying the force-displacement curves were also generated for each set of specimens, with teeth prepared without a finish line (Figure 11) and with a finish line (Figure 12), specifically for the zirconia dental crowns.

**Figure 13:**
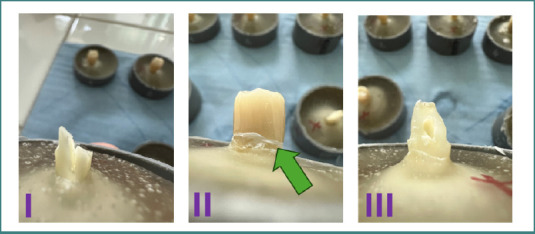
Images of the specimens after the compression test

- Specimens I and III exhibited major changes upon initial inspection, showing horizontal fractures originating from the cervical area (Figure 13).
- Specimen II showed no detectable damage to the dental crown; it is possible that during testing, the crown achieved better seating on the prepared tooth, resulting in only micro-movements. This specimen belongs to the group that recorded lower force values (Figure 13).
- Specimens 1, 2, and 3 did not exhibit significant changes.

The maximum forces at which an event was recorded during the compression tests varied considerably, ranging from 180 to 2537 N.

## DISCUSSION

According to studies published in the specialized literature, the maximum occlusal force in natural dentition is approximately 450 N [8-10]. Thus, it can be observed that, except for Sample II (405 N), all other samples recorded higher values. Other research indicates that during mastication, average compressive forces range between 70 and 150 N, depending on the consistency of the food and the individual’s muscular strength [11,12].

For a more comprehensive interpretation of the obtained results, a statistical analysis of the different preparation types is necessary [13]. However, such an analysis must take into account various factors related to the material properties, the preparation techniques employed, and the manufacturing processes of prosthetic medical devices, as highlighted previously [14]. The specialized literature confirms the superior biomechanical behavior of crowns fabricated from zirconia [15]. Recent analyses have demonstrated that these restorations exhibit significantly higher fracture resistance compared to pressed ceramics, reaching average compressive values between 1650 and 2300 N, depending on abutment design and fixation conditions [16-20]. For instance, in a comparative study conducted by Sorrentino *et al*., zirconia crowns demonstrated an average fracture resistance exceeding 1655 N, compared to 1400 N for all-ceramic crowns [21]. Another study, published in BMC Oral Health in 2024, reported average fracture forces of 2248 N for zirconia crowns on maxillary premolars and 2050 N for mandibular premolars—values significantly higher than those observed in physiological occlusion [22].

These findings support the conclusion that the type of restorative material and the preparation technique directly influence the mechanical performance of the crown. Teeth prepared with a shoulder margin generally exhibited higher maximum force values compared to those prepared without a shoulder, corroborating the findings in the literature regarding the efficiency of the marginal support in preventing fractures [23].

Regarding the type of preparation, a comparative study evaluated five preparation designs: shoulder, shoulderless, pronounced deep chamfer, slight chamfer, and beveled shoulder. The results demonstrated that the shoulder preparation exhibited the highest fracture resistance (≈2286 N), followed by shoulderless (≈2041 N), beveled shoulder (≈1722 N), pronounced chamfer (≈1752 N), and slight chamfer (≈1624 N). Statistically, these differences were significant (ANOVA, *P* < 0.01) [24-26]. In conclusion, shoulder preparation is recommended whenever feasible, which agrees with the present study. Other studies support the notion that a shoulder finish line provides adequate space for the restorative material and ensures optimal marginal adaptation, contributing to uniform distribution of occlusal stresses [26].

Regarding the material used, zirconia is frequently employed in crown restorations due to its exceptional mechanical, aesthetic, and biological properties [27]. Recent studies highlight the following clinical and experimental advantages: zirconia demonstrates significantly higher flexural strength and fracture resistance. Experimental research has shown that the fracture resistance of monolithic zirconia crowns may range between 2000 and 4700 N, depending on material thickness, marginal design, degree of translucency, and the effect of simulated hydrothermal aging cycles [28-31]. These values considerably exceed the threshold of physiological forces observed in natural occlusion, which are estimated at approximately 450 N in the molar region, as also evidenced in the present study [32].

Therefore, after excluding all sample values that experienced adverse events during testing (fractures, detachments, or micromovements)—namely, Sample II and the lowest value recorded for teeth prepared without a shoulder and restored with monolithic zirconia (Sample I)—the remaining values are presented in Table 2. The excluded values are highlighted in red.

**Table 2:** Maximum forces and their processed values

No.	Sample	Maximum force (N)	Maximum force average / Maximum force (N)
1	1	**1091**	2386
2	2	2239	
3	3	2533	
4	I	2301	2419
5	II	**405**	
6	III	2537	

## CONCLUSION

From the values obtained, the following conclusions can be drawn:

- The highest forces were recorded in teeth prepared with a finish line.
- High force values were observed in both teeth prepared with and without a finish line.

The study results indicate that tooth preparation significantly influences the tensile strength of prosthetic crowns. Full-contour zirconia crowns demonstrated maximum strength values on both finish line and non-finish line preparations, with a slight advantage observed for the finish line preparations. These findings suggest that both the type of material used for the crowns and the tooth preparation design impact the durability and stability of fixed prosthetic restorations.

## Personal thanks

We thank 3Dentasoft Dental Laboratory (Bucharest, Romania) for essential support. All zirconia crowns and auxiliary components used in the experimental setup were fabricated in collaboration with the laboratory. We are grateful to the technical team, laboratory manager Cristian Neagu, and especially dental technician Armand Caragea for their skilled work on the prosthetic components. We also thank Linda Radwan for supervising the project and critically reviewing the manuscript for accuracy.
